# The Association between *NQO1* Pro187Ser Polymorphism and Bladder Cancer Susceptibility: A Meta-Analysis of 15 Studies

**DOI:** 10.1371/journal.pone.0116500

**Published:** 2015-01-20

**Authors:** Sen Yang, Tao Jin, Hong-Xia Su, Jin-Hong Zhu, Da-Wen Wang, Shi-Jian Zhu, Sheng Li, Jing He, Ying-He Chen

**Affiliations:** 1 Department of Urology, the Second Affiliated Hospital of Wenzhou Medical University, Wenzhou, Zhejiang, China; 2 State Key Laboratory of Oncology in South China, Department of Experimental Research, Collaborative Innovation Center for Cancer Medicine, Sun Yat-Sen University Cancer Center, Guangzhou, Guangdong, China; 3 Clinical Laboratory, The First People’s Hospital of Yongkang, Yongkang, Zhejiang, China; 4 Department of Molecular Epidemiology and Laboratory Medicine, Harbin Medical University Cancer Hospital, Harbin, Heilongjiang, China; Baylor College of Medicine, UNITED STATES

## Abstract

**Background:**

NAD(P)H:quinone oxidoreductase 1 (NQO1), an obligate two-electron reductase, plays an important role in reducing reactive quinones to less reactive and less toxic hydroquinones. Genetic variations in NQO1 gene that impede its enzyme function may be considered as putative risk factor for cancer. Numerous studies have been performed to investigate the association between NQO1 Pro187Ser polymorphism and bladder cancer risk; nevertheless, the results remain controversial.

**Methods:**

We indentified eligible publications from PubMed, Embase and CBM databases. Pooled odds ratios (ORs) and 95% confidence intervals (CIs) were used to access the strength of the associations. False-positive report probability (FPRP) analysis was also performed for all statistically significant findings.

**Results:**

We collected a total of 15 studies including 4298 cases and 4275 controls in the final meta-analysis. Overall, the NQO1 187Ser carriers were associated with an increased bladder cancer risk (homozygous: OR = 1.43, 95% CI = 1.08-1.90; recessive: OR = 1.33, 95% CI = 1.03-1.72; dominant: OR = 1.19, 95% CI = 1.04-1.37, and allele comparing: OR = 1.18, 95% CI = 1.06-1.33). Stratification analyses showed a statistically significant association among Asians (homozygous: OR = 1.82, 95% CI = 1.39-2.38; recessive: OR = 1.52, 95% CI = 1.20-1.93, dominant: OR = 1.40, 95% CI = 1.05-1.88, and allele comparing: OR = 1.35, 95% CI = 1.15-1.58), never smokers (homozygous: OR = 2.30, 95% CI = 1.14-4.65; heterozygous: OR = 2.26, 95% CI = 1.43-3.56; dominant model: OR = 1.59, 95% CI = 1.14-2.21, and allele comparing: OR = 1.72, 95% CI = 1.27-2.33), hospital-based studies (homozygous: OR = 1.46, 95% CI = 1.09-1.94; recessive: OR = 1.32, 95% CI = 1.02-1.69; dominant: OR = 1.28, 95% CI = 1.05-1.56, and allele comparing: OR = 1.24, 95% CI = 1.07-1.43), studies with genotyping performed by PCR-RFLP under all genetic models, and studies with minor allele frequency >0.30 (homozygous: OR = 1.69, 95% CI = 1.25-2.27; recessive: OR = 1.46, 95% CI = 1.10-1.95, and allele comparing: OR = 1.25, 95% CI = 1.04-1.51), respectively.

**Conclusions:**

Despite some limitations, our meta-analysis provides sufficient evidence that NQO1 Pro187Ser polymorphism may contribute to bladder cancer risk. These findings need further validation in well-designed prospective studies with larger sample size and different ethnicities, especially for Asians.

## Introduction

Cancer is a major public health burden worldwide. Approximately 12.7 million new cancer cases and 7.6 million cancer deaths were reported based on GLOBOCAN 2008 [1]. Bladder cancer is the seventh most frequently diagnosed cancer among men, with about 386300 new cancer cases and 150200 cancer deaths in 2008 [1]. It is clear that cancer is a multistep process involving complex interactions between environmental and genetic factors [2,3]. Cigarette smoking, occupational exposure to chemical carcinogens, drugs consuming (e.g., phenacetin and cyclophosphamide), as well as *Schistosoma hematobium* infection are well-known etiologic factors for bladder cancer [4]. Despite the environmental factors play important roles in the development of cancer, host genetic factors are also closely related to the pathophysiology of many human cancers including bladder cancer [5].

NAD(P)H:quinone oxidoreductase 1 (NQO1), also known as diphtheria toxin diaphorase (DT-diaphorase), is a cytoplasmic 2-electron reductase, belonging to the NAD(P)H dehydrogenase (quinone) family. NQO1 plays an important role in the aromatic amine metabolism pathway [6]. It acts to reduce and detoxifies quinines and their derivative, thereby protecting cells against oxidative stress and carcinogenesis. Polymorphisms that lead to aberrant alteration of NQO1 activity may have profound impacts on cancer disposition.

The *NQO1* gene is mapped to chromosome 16q22.1 and comprised of six exons and five introns [7,8]. Among all the identified single nucleotide polymorphisms (SNPs) in this gene, a non-synonymous *NQO1* rs1800566 SNP is of particular interest and widely investigated in molecular epidemiology studies. This SNP is located at nucleotide position 609, causing a C-to-T transition and consequential proline to serine amino acid substitution at codon 187 in exon six [8]. Genotype-phenotype studies have demonstrated that the rs1800566 C>T (Pro187Ser) polymorphism is associated with a decreased activity of NQO1 enzymatic activity [9,10]. Compared with the wild-type CC genotype carriers, the homozygous TT genotype carriers have only 2–4% of the quinone reductase activity [9,10]. As expected, it has been reported that the polymorphism in the xenobiotic metabolizing enzyme gene, *NQO1* Pro187Ser, is associated with bladder cancer risk [11]. Besides this study, numerous studies have investigated the association between *NQO1* Pro187Ser polymorphism and bladder cancer risk [12–32], however, the conclusions remain controversial rather than conclusive. With this in mind, we carried out the current meta-analysis and aimed to investigate the association between this polymorphism and bladder cancer susceptibility, and to draw a more precise conclusion. The false-positive report probability (FPRP) analysis was also conducted to preclude false association resulting from multiple calculations.

## Materials and Methods

### Literature search strategy

Literatures examining the association between *NQO1* Pro187Ser polymorphism and bladder cancer risk were searched from MEDLINE and EMBASE databases using the following key words: “NQO1 or NAD(P)H:quinone oxidoreductase 1 or DT-diaphorase or DTD or quinone reductase or rs1800566 C>T or Pro187Ser”, “polymorphism or variant or variation” and “bladder” (prior to May 16, 2014). We also searched additional related publications on this topic from the literature cited by each article identified from the mentioned database. We only included the latest or the largest study in our final meta-analysis when the studies with overlapping data published by same authors or from the same institutions. As native-Chinese speakers, we also searched publications written in Chinese from Chinese Biomedical (CBM) database with the combinations of “*NQO1*” and “bladder cancer” to maximize the coverage of our investigation and minimize the selection bias.

### Inclusion and exclusion criteria

Studies included should meet the following inclusion criteria: (1) evaluating the association between *NQO1* Pro187Ser polymorphism and bladder cancer risk; (2) case-control design; (3) sufficient data including allele frequency and distribution of genotypes provided to estimate odds ratios (ORs) and their 95% confidence intervals (CIs); (4) genotype distribution in the controls was consistent with Hardy–Weinberg equilibrium (HWE); (5) independent from other studies.

Studies would be excluded if they were review articles, case only or non-cancer subjects only studies, conference abstracts, case reports, studies deviated from HWE in controls, and duplicate publications.

### Data extraction

Two authors (Sen Yang and Tao Jin) extracted the following information independently from each publication with the inclusion criteria mentioned above: the first author’s surname, publication year, country of origin, ethnicity, method of matching controls to cases, source of controls, smoking status, genotyping methods, total number of cases and controls, numbers of cases and controls with the CC (Pro/Pro), CT (Pro/Ser) and TT (Ser/Ser) genotypes, HWE and minor allele frequency (MAF) for controls. We conducted stratification analyses by ethnicity (categorized as Caucasians, Asians and Africans), source of control (hospital-based and population-based), genotyping methods [restriction fragment length polymorphism PCR (PCR-RFLP), mass spectrometry and TaqMan], MAF (<0.20, 0.20–0.30 and >0.30 according to the frequency and number of each investigation), and smoking status (never smokers and ever smokers). Any disagreements were resolved by discussions between the two authors until consensus reached.

### Statistical methods

The association between *NQO1* Pro187Ser polymorphism and bladder cancer risk was evaluated by homozygous model (Ser/Ser vs. Pro/Pro), heterozygous model (Pro/Ser vs. Pro/Pro), recessive model [Ser/Ser vs. (Pro/Ser + Pro/Pro)], dominant model [(Pro/Ser + Ser/Ser) vs. Pro/Pro] as well as allele comparison (Ser vs. Pro). Goodness-of-fit chi-square test was used to test deviation from HWE and *P*<0.05 was considered as departure from HWE. Chi square-based Q-test was used to evaluate the between-study heterogeneity. Effect of heterogeneity was also quantified with the I^2^ statistic which measures the degree of between-study inconsistency. I^2^ lies between 0 to 100% with higher values indicating a greater degree of heterogeneity [33]. Fixed-effects model (Mantel-Haenszel method) were chosen when the *P* value of the heterogeneity test was ≥ 0.10 or I^2^ < 50% [34]; otherwise, the random-effects model (DerSimonian and Laird method) were selected which tends to provide wider 95% CIs [35]. Potential publication bias was verified by standard error of log (OR) for each study plotted against their consequential log (OR). Egger’s linear regression test was used to assess the Funnel plot asymmetry [36,37]. Sensitivity analyses were calculated by removing a single study individually and recalculating the corresponding ORs and 95% CIs.

It has been acknowledged that many reported associations between genetic variation and susceptibility to diseases might be false positive, given determination of statistical significance largely relies on *P* value alone. To rule out false associations resulted from multiple tests, false-positive report probability (FPRP) analysis was also performed for all statistically significant findings as described previously [38–40]. Briefly, FPRP value for a given association between SNP of interest and bladder cancer risk was calculated with FPRP threshold of 0.2 and prior probability of 0.1. Statistical power was used to detect an OR of 1.50/0.67 for alleles with a risk/protective effect. An association with FPRP value below 0.2 was declared noteworthy. Meta-regression analysis was performed to investigate the major sources of heterogeneity across the studies in the current study.

STATA (version 11.0; Stata Corporation, College Station, TX) and SAS software (version 9.1; SAS Institute, Cary, NC) were used for statistical analyses. All *P* values were two-sided, and *P*<0.05 was considered as statistically significant finding.

## Results

### Characteristics of study

**Figure 1:**
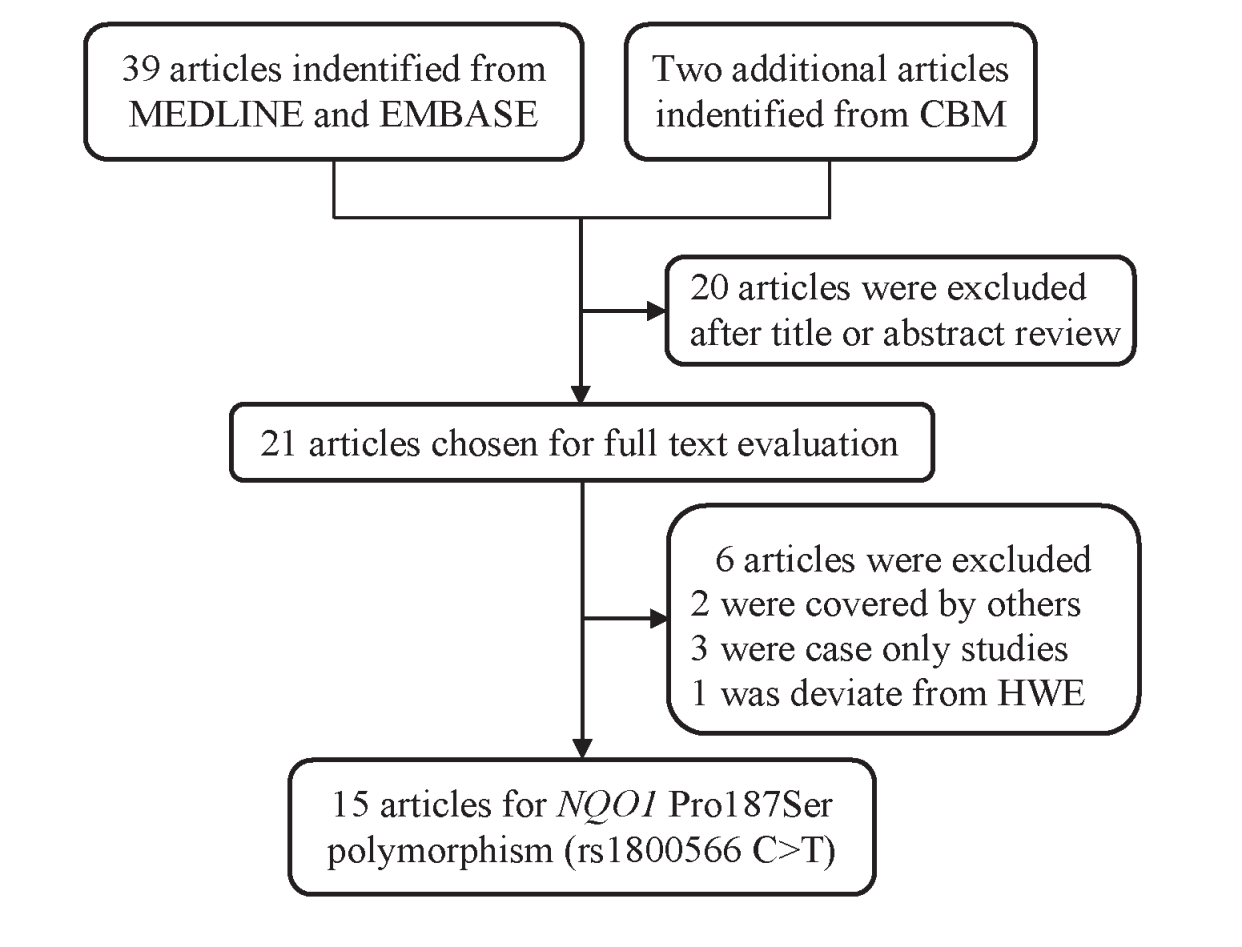
Flow chart of included studies for the association between *NQO1* Pro187Ser polymorphism.

A total of 39 publications examining the association between *NQO1* Pro187Ser and bladder cancer risk were identified from MEDLINE and EMBASE, and two additional publications from CBM (Fig. 1). Initial screening led to the removal of 20 articles for failure to meet eligibility requirements. As a result, 21 studies that fulfilled the crude inclusion criteria were chosen for further analysis [12–32]. Among them, two [28,29] were excluded, one was overlapped with a study published more recently [24], and the other duplicated the use of subjects that were reported in a study with much larger sample size [25]. Additional three studies were excluded from our final analysis due to reporting case only studies [30–32]. In the last, one study was also excluded for the deviation from HWE (*P*<0.001) in controls [23]. Ultimately, 15 publications with a total of 4298 bladder cancer cases and 4275 controls were included in the final meta-analysis (Table 1). In the included investigations, sample sizes ranged from 61 to 1128 for bladder cancer cases and from 100 to 1123 for controls. There were eight studies conducted on Caucasians, six studies on Asians, and only one study on Africans. In term of study design, five studies were population-based, and ten were hospital-based. Moreover, eight studies provided the genotype information for ever smokers and never smokers, respectively (S1 Table). Genotyping methods used in the studies included PCR-RFLP (11 studies), TaqMan (two studies) and mass spectrometry (two studies).

**Table 1:** Characteristics of studies included in the meta-analysis for an association between *NQO1* Pro187Ser (1800566 C>T) polymorphism and bladder cancers risk.

Surname	Year	Country	Ethnicity	Source	Genotype method	Case				Control				MAF	HWE
						CC	CT	TT	All	CC	CT	TT	All		
Schulz	1997	Germany	Caucasian	PB	PCR-RFLP	68	26	8	102	195	61	4	260	0.13	0.755
Park	2003	USA	Caucasian	HB	PCR-RFLP	142	82	8	232	163	66	10	239	0.18	0.321
Choi	2003	Korea	Asian	HB	PCR-RFLP	81	68	28	177	64	90	16	170	0.36	0.050
Sanyal	2004	Sweden	Caucasian	PB	PCR-RFLP	206	85	8	299	83	34	7	124	0.19	0.175
Moore	2004	Argentina	Caucasian	PB	PCR-RFLP	62	35	9	106	61	40	7	108	0.25	0.898
Hung	2004	Italy	Caucasian	HB	PCR-RFLP	113	75	13	201	135	66	13	214	0.21	0.207
Terry	2005	USA	Caucasian	HB	Mass spectrometry	156	70	9	235	150	58	6	214	0.16	0.890
Broberg	2005	Sweden	Caucasian	PB	Mass spectrometry	43	13	5	61	107	46	3	156	0.17	0.442
Wang	2008	China	Asian	HB	PCR-RFLP	70	148	82	300	94	136	70	300	0.46	0.130
Figueroa	2008	Spain	Caucasian	HB	TaqMan	685	392	51	1128	661	400	62	1123	0.23	0.884
Pandith	2011	India	Asian	HB	PCR-RFLP	44	53	7	104	70	46	4	120	0.23	0.277
Fu	2013	China	Asian	HB	PCR-RFLP	30	38	31	99	38	46	16	100	0.39	0.740
Huang	2014	China	Asian	HB	PCR-RFLP	36	83	40	159	51	67	32	150	0.44	0.259
Goerlitz	2014	Egypt	Africa	PB	TaqMan	519	323	53	895	470	276	51	797	0.24	0.226
Mandal	2014	India	Asian	HB	PCR-RFLP	105	72	23	200	128	61	11	200	0.21	0.304

HB, Hospital based; PB, Population based; PCR-RFLP, Polymorphism chain reaction- restriction fragment length polymorphism; MAF, Minor allelic frequency; HWE, Hardy-Weinberg equilibrium

### Meta-analysis results

**Figure 2:**
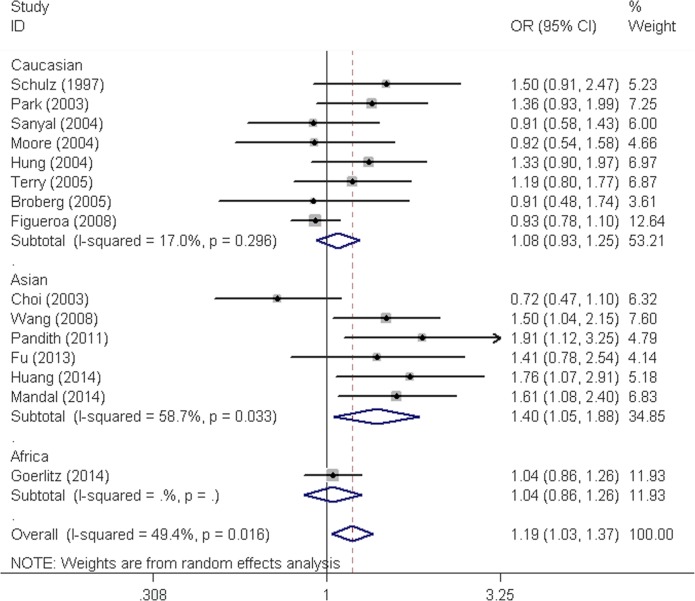
Forest plot for *NQO1* Pro187Ser polymorphism and bladder cancer risk under the dominant model by ethnicity. For each study, the estimates of OR and its 95% CI are plotted with a box and a horizontal line. Diamond indicates pooled ORs and its 95% CIs.

As shown in Table 2 and Fig. 2, when all the eligible studies were pooled together, analyses yielded significant associations between the variant of interest and the risk of bladder cancer. Carriers of variant Ser allele in codon 187 of *NQO1* exhibited significant increased risk for bladder cancer, when compared to non-carriers (homozygous model: OR = 1.43, 95% CI = 1.08–1.90; recessive model: OR = 1.33, 95% CI = 1.03–1.72; dominant model: OR = 1.19, 95% CI = 1.04–1.37, and allele comparing: OR = 1.18, 95% CI = 1.06–1.33). Stratification analysis by ethnicity revealed a statistically significant association with bladder cancer risk among Asians (homozygous model: OR = 1.82, 95% CI = 1.39–2.38; recessive model: OR = 1.52, 95% CI = 1.20–1.93; dominant model: OR = 1.40, 95% CI = 1.05–1.88, and allele comparing: OR = 1.35, 95% CI = 1.15–1.58), but not among Caucasian. When stratified by the source of control, an increased risk of bladder cancer was also suggested for variant alleles of *NQO1* Pro187Ser polymorphism in the hospital-based subgroup (homozygous model: OR = 1.46, 95% CI = 1.09–1.94; recessive model: OR = 1.32, 95% CI = 1.02–1.69; dominant model: OR = 1.28, 95% CI = 1.05–1.56, and allele comparing: OR = 1.24, 95% CI = 1.07–1.43), while no effect was observed in the population-based subgroup. When studies were stratified by the genotyping methods, we observed an increased bladder cancer risk among studies using PCR-RFLP method (homozygous: OR = 1.61, 95% CI = 1.20–2.18; heterozygous: OR = 1.23, 95% CI = 1.01–1.49; recessive: OR = 1.46, 95% CI = 1.09–1.95; dominant: OR = 1.30, 95% CI = 1.09–1.55, and allele comparing: OR = 1.27, 95% CI = 1.12–1.44). We also found that there was a statistically significant association between increased risk and the SNP of interest (homozygous: OR = 1.69, 95% CI = 1.25–2.27; recessive: OR = 1.46, 95% CI = 1.10–1.95, and allele comparing: OR = 1.25, 95% CI = 1.04–1.51) among studies with MAF>0.30. Additionally, we found that the risk of bladder cancer associated with *NQO1* Pro187Ser polymorphism was pronounced and statistically significant among never smokers (homozygous model: OR = 2.30, 95% CI = 1.14–4.65; heterozygous model: OR = 2.26, 95% CI = 1.43–3.56; dominant model: OR = 1.59, 95% CI = 1.14–2.21, and allele comparing: OR = 1.72, 95% CI = 1.27–2.33), but no association was detected when smokers were considered.

**Table 2:** Meta-analysis of the association between *NQO1* Pro187Ser (1800566 C>T) polymorphism and bladder cancer risk.

Variables	No. of studies	Case/Control	Homozygous			Heterozygous			Recessive			Dominant			Allele Comparing		
			TT vs. CC			CT vs. CC			TT vs. (CT + CC)			(CT + TT) vs. CC			T vs. C		
			OR (95% CI)	*P* ^het^	*I* ^2^	OR (95% CI)	*P* ^het^	*I* ^2^	OR (95% CI)	*P* ^het^	*I* ^2^	OR (95% CI)	*P* ^het^	*I* ^2^	OR (95% CI)	*P* ^het^	*I* ^2^
All	15	4298/4275	1.43 (1.08–1.90)	0.009	52.8	1.14 (0.99–1.31)	0.034	44.1	1.33 (1.03–1.72)	0.015	49.8	1.19 (1.04–1.37)	0.016	49.4	1.18 (1.06–1.33)	0.008	53.3
Ethnicity																	
Caucasian	8	2364/2438	1.24 (0.77–2.01)	0.029	55.3	1.05 (0.92–1.21)	0.389	5.3	1.21 (0.75–1.96)	0.028	55.4	1.08 (0.93–1.25)	0.296	17.0	1.10 (0.94–1.28)	0.110	40.3
Asian	6	1039/1040	1.82 (1.39–2.38)	0.736	0.0	1.27 (0.90–1.79)	0.011	66.5	1.52 (1.20–1.93)	0.414	0.3	1.40 (1.05–1.88)	0.033	58.7	1.35 (1.15–1.58)	0.201	31.3
African	1	895/797	0.94 (0.63–1.41)	/	/	1.06 (0.87–1.30)	/	/	0.92 (0.62–1.37)	/	/	1.04 (0.86–1.26)	/	/	1.01 (0.87–1.19)	/	/
Source of control																	
PB	5	1463/1445	1.48 (0.68–3.22)	0.010	70.0	1.03 (0.87–1.21)	0.743	0.0	1.49 (0.68–3.28)	0.008	71.1	1.04 (0.89–1.22)	0.602	0.0	1.08 (0.88–1.32)	0.165	38.5
HB	10	2835/2830	1.46 (1.09–1.94)	0.081	41.5	1.22 (1.00–1.49)	0.008	59.5	1.32 (1.02–1.69)	0.137	33.9	1.28 (1.05–1.56)	0.005	62.3	1.24 (1.07–1.43)	0.008	59.6
Genotyping method																	
PCR-RFLP	11	1979/1985	1.61 (1.20–2.18)	0.126	34.0	1.23 (1.01–1.49)	0.057	44.2	1.46 (1.09–1.95)	0.090	38.8	1.30 (1.09–1.55)	0.076	40.9	1.27 (1.12–1.44)	0.125	34.2
MS	2	296/370	2.15 (0.79–5.86)	0.254	23.3	0.99 (0.62–1.56)	0.232	29.9	2.24 (0.71–7.06)	0.194	40.8	1.10 (0.79–1.55)	0.500	0.0	1.17 (0.88–1.57)	0.966	0.0
TaqMan	2	2023/1920	0.86 (0.65–1.14)	0.550	0.0	0.99 (0.87–1.13)	0.406	0.0	0.86 (0.66–1.13)	0.649	0.0	0.97 (0.86–1.11)	0.367	0.0	0.96 (0.87–1.07)	0.374	0.0
MAF																	
<0.20	5	929/993	1.58 (0.65–3.84)	0.016	67.2	1.15 (0.93–1.43)	0.503	0.0	1.54 (0.63–3.80)	0.012	68.9	1.19 (0.97–1.45)	0.512	0.0	1.18 (0.96–1.46)	0.217	30.7
0.20–0.30	6	2634/2562	1.20 (0.82–1.77)	0.077	49.6	1.14 (0.95–1.37)	0.098	46.2	1.10 (0.80–1.51)	0.191	32.7	1.17 (0.95–1.45)	0.024	61.3	1.15 (0.96–1.39)	0.009	67.2
>0.30	4	735/720	1.69 (1.25–2.27)	0.719	0.0	1.12 (0.69–1.84)	0.008	74.8	1.46 (1.10–1.95)	0.313	15.7	1.27 (0.84–1.90)	0.024	68.2	1.25 (1.04–1.51)	0.220	32.1
Smoking status																	
Ever	8	757/592	1.19 (0.70–2.03)	0.810	0.0	1.00 (0.65–1.54)	0.102	51.7	1.11 (0.67–1.83)	0.945	0.0	1.21 (0.82–1.77)	0.001	71.0	1.04 (0.83–1.29)	0.385	1.5
Never	8	295/484	2.30 (1.14–4.65)	0.720	0.0	2.26 (1.43–3.56)	0.602	0.0	1.44 (0.79–2.64)	0.603	0.0	1.59 (1.14–2.21)	0.130	37.5	1.72 (1.27–2.33)	0.536	0.0

HB, Hospital based; PB, Population based; PCR-RFLP, Polymorphism chain reaction- restriction fragment length polymorphism; MS, Mass spectrometry; MAF, Minor allelic frequency

The FPRP values for all statistically significant result are shown in Table 3. For a prior probability of 0.1 and OR of 1.5, the FPRP analysis indicated that the significant association was noteworthy for all subjects (homozygous: FPRP = 0.098, dominant: FPRP = 0.119, and allele comparison: FPRP = 0.034), Asians (homozygous: FPRP = 0.001, recessive: FPRP = 0.016, dominant: FPRP = 0.173, and allele comparison: FPRP = 0.002), HB designed studies (homozygous: FPRP = 0.092, dominant: FPRP = 0.112, and allele comparison: FPRP = 0.035), studies using PCR-RFLP method for genotyping (homozygous: FPRP = 0.029, recessive: FPRP = 0.097, dominant: FPRP = 0.188, and allele comparison: FPRP = 0.002) and studies with MAF >0.30 (homozygous: FPRP = 0.035, recessive: FPRP = 0.101, and allele comparison: FPRP = 0.126). Likewise, association among never smokers was also deserving of attention (heterozygous: FPRP = 0.059, dominant: FPRP = 0.068, and allele comparison: FPRP = 0.032). Relatively greater FPRP values were found for other noteworthy findings between *NQO1* Pro187Ser polymorphism and bladder cancer risk may be due to the limited sample sizes, which need further validation in the studies with large sample size.

**Table 3:** False-positive report probability values for associations between bladder cancer risk and the frequency of genotypes of *NQO1* gene.

Variables	Case/Control	OR (95% CI)	*P* ^a^	Statistical Power^b^	Prior Probability				
					0.25	0.1	0.01	0.001	0.0001
Homozygous (TT vs. CC)									
All	2735/2782	1.43 (1.08–1.90)	0.012	0.997	0.035	0.098	0.544	0.923	0.992
Asian	577/594	1.82 (1.39–2.38)	0.00002	0.196	0.000	0.001	0.008	0.075	0.450
HB	1754/1794	1.46 (1.09–1.94)	0.011	0.980	0.033	0.092	0.526	0.918	0.991
PCR-RFLP	1214/1272	1.61 (1.20–2.18)	0.002	0.595	0.01	0.029	0.250	0.771	0.971
MAF >0.30	398/381	1.69 (1.25–2.27)	0.001	0.245	0.012	0.035	0.288	0.803	0.976
Never smoker	199/391	2.30 (1.14–4.65)	0.020	0.177	0.253	0.504	0.918	0.991	0.999
Heterozygous (CT vs. CC)									
PCR-RFLP	1722/1795	1.23 (1.01–1.49)	0.038	1.000	0.102	0.255	0.790	0.974	0.997
Never smoker	250/455	2.26 (1.43–3.56)	0.0004	0.064	0.021	0.059	0.410	0.875	0.986
Recessive [TT vs. (CT + CC)]									
All	4298/4275	1.33 (1.03–1.72)	0.030	0.999	0.083	0.213	0.748	0.968	0.997
Asian	1039/1040	1.52 (1.20–1.93)	0.001	0.568	0.005	0.016	0.148	0.638	0.946
HB	2835/2830	1.32 (1.02–1.69)	0.032	0.993	0.088	0.225	0.761	0.970	0.997
PCR-RFLP	1979/1985	1.46 (1.09–1.95)	0.011	0.921	0.035	0.097	0.542	0.923	0.992
MAF >0.30	735/720	1.46 (1.10–1.95)	0.009	0.717	0.036	0.101	0.554	0.926	0.992
Dominant [(CT + TT) vs. CC]									
All	4298/4275	1.19 (1.04–1.37)	0.015	1.000	0.043	0.119	0.598	0.937	0.993
Asian	1039/1040	1.40 (1.05–1.88)	0.023	0.986	0.065	0.173	0.698	0.959	0.996
HB	2835/2830	1.28 (1.05–1.56)	0.014	1.000	0.040	0.112	0.581	0.933	0.993
PCR-RFLP	1979/1985	1.30 (1.09–1.55)	0.024	0.934	0.072	0.188	0.718	0.962	0.996
Never smoker	295/484	1.59 (1.14–2.21)	0.006	0.742	0.024	0.068	0.445	0.890	0.988
Allele (T vs. C)									
All	8596/8550	1.18 (1.06–1.23)	0.011	1.000	0.012	0.034	0.280	0.797	0.975
Asian	2078/2080	1.35 (1.15–1.58)	0.0002	0.997	0.001	0.002	0.024	0.198	0.712
HB	5670/5660	1.24 (1.07–1.43)	0.004	1.000	0.012	0.035	0.286	0.801	0.976
PCR-RFLP	3958/3970	1.27 (1.12–1.44)	0.0002	1.000	0.001	0.002	0.022	0.184	0.693
MAF >0.30	1470/1440	1.25 (1.04–1.51)	0.016	0.998	0.046	0.126	0.613	0.941	0.994
Never smoker	590/968	1.72 (1.27–2.33)	0.001	0.272	0.011	0.032	0.267	0.786	0.974

CI, confidence interval; OR, odds ratio; HB, Hospital based

^a^ Chi-square test was used to calculate the genotype frequency distributions

^b^ Statistical power was calculated using the number of observations in the subgroup and the OR and *P* values in this table

### Heterogeneity and sensitivity analyses

Moderate heterogeneities were found for the studies assessing the association between *NQO1* Pro187Ser polymorphism and bladder cancer risk (homozygous model: *P* = 0.009, I^2^ = 52.8%; heterozygous model: *P* = 0.034, I^2^ = 44.1%; recessive model: *P* = 0.015, I^2^ = 49.8%; dominant model: *P* = 0.016, I^2^ = 49.4%, and allele comparing: *P* = 0.008, I^2^ = 53.3%). Therefore, the random-effects model was chosen since it generates wider CIs. Moreover, while the leave-one-out sensitivity analysis was performed, none of any single study altered the pooled ORs qualitatively (data not shown).

With the purpose to find the source of heterogeneity, we also performed meta-regression by ethnicity, source of controls, genotyping methods and MAF. As shown in Table 4, we found that source of controls might contributed to the heterogeneity in the current meta-analysis to some extent although *P* values were not statistical significant (heterozygous model: *P* = 0.067; recessive model: *P* = 0.096). In contrast, other covariates were not likely to cause heterogeneity across studies as indicated by *P* values: ethnicity (homozygous model: *P* = 0.866; heterozygous model: *P* = 0.109; dominant model: *P* = 0.969; recessive model: *P* = 0.160 and allele comparing model: *P* = 0.284), genotyping method (homozygous model: *P* = 0.842; heterozygous model: *P* = 0.097; dominant model: *P* = 0.988; recessive model: *P* = 0.107 and allele comparing model: *P* = 0.172), and MAF (homozygous model: *P* = 0.970; heterozygous model: *P* = 0.084; dominant model: *P* = 0.830; recessive model: *P* = 0.160 and allele comparing model: *P* = 0.289).

**Table 4:** Meta-regression analysis of the main characteristics for the 15 studies.

Variables	Homozygous			Heterozygous			Dominant			Recessive			Allele comparing		
	Coef.	95% CI	*P*	Coef.	95% CI	*P*	Coef.	95% CI	*P*	Coef.	95% CI	*P*	Coef.	95% CI	*P*
Ethnicity	0.06	(-0.76, 0.89)	0.866	0.23	(-0.06, 0.51)	0.109	-0.01	(-0.83, 0.80)	0.969	0.20	(-0.09, 0.49)	0.160	0.12	(-0.12, 0.38)	0.284
Control source	0.06	(-1.04, 1.16)	0.905	0.38	(-0.03, 0.79)	0.067	-0.08	(-1.16, 1.01)	0.880	0.33	(-0.07, 0.73)	0.096	0.21	(-0.13, 0.56)	0.201
Genotype method	-0.06	(-0.77, 0.64)	0.842	-0.20	(-0.44, 0.04)	0.097	0.01	(-0.69, 0.70)	0.988	-0.19	(-0.43, 0.05)	0.107	-0.14	(-0.35, 0.07)	0.172
MAF	0.02	(-0.86, 0.89)	0.970	-0.28	(-0.60, 0.05)	0.084	-0.08	(-0.77, 0.94)	0.830	-0.22	(-0.54, 0.10)	0.160	-0.14	(-0.41, 0.14)	0.289

MAF, Minor allelic frequency

### Publication bias

**Figure 3:**
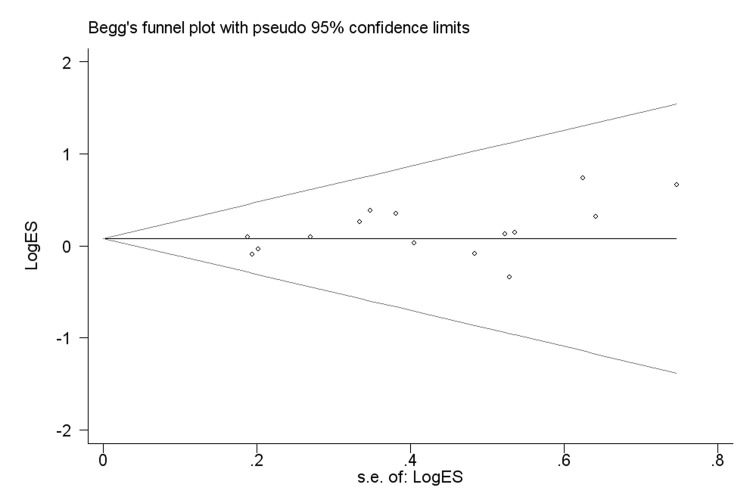
Funnel plot analysis to detect publication bias for *NQO1* Pro187Ser polymorphism under recessive model. Each point represents a separate study for the indicated association.

We performed both Begg’s and Egger’s tests to evaluate the publication bias for the studies included in the final meta-analysis (Fig. 3). The shapes of the funnel plots seemed symmetrical, indicating no significantly statistical evidence of publication bias for the association between *NQO1* Pro187Ser polymorphism and bladder cancer risk (data not shown).

## Discussion

In the current updated meta-analysis of 15 studies including a total of 4298 cases and 4275 controls, we found that the *NQO1* Pro187Ser polymorphism was associated with increased bladder cancer susceptibility. Further stratified analyses revealed that this association was more predominant among Asians, hospital-based studies, studies adopting PCR-RFLP method for genotyping, studies with MAF >0.30, as well as never smokers. Noteworthily, these results were further confirmed by FPRP analysis.

To our knowledge, several meta-analyses have been performed to investigate the role of *NQO1* Pro187Ser polymorphism in the bladder cancer risk [41–45]. In a meta-analysis of eight studies including 1410 cases and 1485 controls [41], Chao et al. observed increased bladder cancer risk with homozygous rare Ser/Ser genotype for the white. After having pooled together 12 studies with 3041 cases and 3128 controls [42], Gao et al. found the significant association only under the allele comparing, but not other genetics models. The study carried out by Zhang et al. [43], including nine publications with 2661 cases and 2738 controls, observed no association under all genetics models. Similarly, Gong et al. also failed to detect any association, while analyzing 11 studies with 2937 cases and 3008 controls [44]. A meta-analysis by Lajin et al. [45], pertaining to all types of cancer, observed a significantly increased overall cancer risk with variant Ser/Ser genotype, as compared to the homozygous wild type (Pro/Pro). When stratified by cancer type, the same study revealed a significantly increased risk of bladder under all five genetics models, involving 10 studies included 1814 bladder cancer cases and 1905 controls. In a meta-analysis carried out by Mandal et al. [27], 12 studies with a total of 2286 cases and 2294 controls were included, they found the Pro187Ser polymorphism was associated with bladder cancer risk under all genetic models. The controversial results in single studies and even meta-analyses urged us to reevaluate the association with inclusion of the latest studies. Some of the previous significant findings were not replicated in our meta-analysis. For example, unlike Chao et al. [41], we did not observe a significant increased risk among Caucasians. We also did not replicate the association between Pro187Ser and bladder cancer risk in heterozygous model reported by Lajin et al. and Mandal et al. [27,45]. However, we did validate some findings from previous publication, i.e. the polymorphism was significantly associated with bladder cancer risk under allele comparison model [42,45], and the association was significant among the Asian group [42]. More importantly, some novel findings were discovered in the current meta-analysis besides. For instance, we observed significantly increased risk of developing bladder cancer among never smokers who carried variant alleles of *NQO1* Pro187Ser polymorphism.

To preclude the possibility that the findings may be false positive due to the limited sample size, FPRP analysis was performed in the current meta-analysis. The associations among all subjects, Asians, studies with hospital-based design, PCR-RFLP genotyping method, MAF >0.30, and never smokers were validated. It is well known that association studies solely depending on a *P* value may sometime lead to false positive findings. To address this issue, FPRP analysis has been introduced, in which the observed *P* value coupled with both the prior probability and the statistical power of the test to validate the significance of a finding [38]. Thus, it is important to perform FPRP analysis to further validate the positive findings and calculated statistical power in a meta-analysis [46]. Theoretically, FPRP analysis with a smaller prior probability would be more likely to determine a real positive finding, Nevertheless, in that case, we might miss some real positive findings due to the limited sample size and weak effect of selected SNPs. Therefore, in the current study, we selected the prior probability of 0.1, which was proper to detect relatively weak effect of SNPs.

NQO1 is an obligate two-electron reductase which can reduce reactive quinones to hydroquinones. Additionally, NQO1 also shows superoxide scavenging activity as well as protective activity against procarcinogenic benzenes, thus it has been considered as an important defense of host against cancer [47,48]. NQO1 plays an important role in protecting cells against chemically induced oxidative stress, cytotoxicity, mutagenicity, and carcinogenicity [14]. It is also implicated in bioactivation of certain environmental procarcinogens (e.g., nitroaromatic compounds and heterocyclic amines) that are present in tobacco smoke and certain processed foods [49,50]. Many molecular epidemiology studies have investigated the association between *NQO1* Pro187Ser polymorphism and bladder cancer risk, but the conclusions were controversial. Some studies reported that the Ser/Ser genotype of Pro187Ser polymorphism was significantly associated with increased bladder cancer risk, when Pro/Pro genotype was used as the reference group [12,21,24], whereas others failed to confirm this finding. Some studies also found the Pro187Ser polymorphism was associated with bladder cancer risk under the dominant model [21,22,26], or under the recessive model [12,18,24]. Overall, the Pro187Ser polymorphism has been shown to significantly associate with an increased bladder cancer risk. The association was also observed among Asians subjects, hospital-based studies, and never smokers. These significant findings may be ascribed to a much larger sample size in the current study than previous meta-analyses. In the current study, we found that the *NQO1* Pro187Ser polymorphism was shown to associate with bladder cancer in hospital-based subgroups but not population-based subgroups. Since the hospital-based subjects are usually less representative and more prone to bias than population-based studies, these results should be interpreted cautiously. We also found the never smokers harboring the variant genotype were associated with increased bladder cancer risk. The main reason may be ascribed to genetic susceptibility. In other words, low penetrance polymorphism, like *NQO1* Pro187Ser, commonly only has weak effect on bladder cancer risk. Therefore, the effect of this SNP among smokers might be superseded by the much stronger effects of smoking. Other reasons for this positive association among never smokers could be the reduced sample sizes in the subgroups, and the fact that the never smokers may also be exposed to second-hand smoke and other risk factors. Furthermore, sensitivity analyses demonstrated that successively excluding one study at a time did not lead to obvious change in the pooled ORs, which strengthens the results of this meta-analysis. Meta-regression suggested that source of controls but not other covariates might partially explain the heterogeneity in the current meta-analysis. In addition, the absence of publication bias further supports the validity of our finding.

Though we have included the latest investigations, some limitations should be acknowledged, while interpreting the results. First, due to lack of original data, our conclusions were based on unadjusted estimates of ORs without adjustment for age, gender, *Schistosoma hematobium* infection status and other risk factors (e.g., smoking and drinking status), which may lead some confounding bias. Second, the controls were not uniformly defined; some studies used healthy subjects as the control group, whereas others used cancer-free patients as the reference group. Third, sample size for each case-control study was relative small, with most studies including less than 500 cases, except for two [20,25]. Therefore, pooled data was not sufficient enough to perform a comprehensive analysis, particularly for subgroup analysis by cancer type and smoking status. Fourth, there was only one study conducted in African population [25], and five studies in Asian subjects. Further studies with large sample size are needed urgently verify our finding, especially those regarding Asians. Fourth, some degree of heterogeneity was found in overall comparison under all genetic models. Heterogeneity is a potential problem when interpreting the results of all meta-analyses [51]. Meta-analysis is a statistical technique to integrate the findings from independent comparable studies on the same topic. A meta-analysis is necessary, if each single study is too small or too restricted in scope to yield conclusive results on a given topic. However, since each study was carried out independently, inter-study variation might be unavoidable, including the source of control, ethnicity, genotyping method, etc. As a result, combining the findings across such studies may suffer from between-study heterogeneity. To overcome this obstacle in integrating various studies, DerSimonian et al. introduced random-effects model in 1986, which can tolerate variations across studies and use an objective method for weighting that can be generalized by including study characteristics into the analysis [35]. In this study, random-effects model was chosen due to heterogeneity. In the subgroup analysis, we found that the heterogeneity might not result from each of factors individually, but from combined resources. For example, as to the ethnicity, Caucasians exhibited heterogeneity under homozygous and recessive models but not other models, whereas Asians showed heterogeneity only under heterozygous and dominant models. Finally, common genetic analysis challenges were only addressed to some extent, including direction of effect of alleles, MAF cutoffs for common and rare variants, technical quality control challenges, and related measures when taking data from lab-generated output to genetic statistical analysis.

In conclusion, the current meta-analysis indicates that the *NQO1* Pro187Ser polymorphism may confer host increased genetic susceptibility to the risk of bladder cancer. However, large and well-designed studies are warranted to validate our findings.

## Supporting Information

S1 Data Original Data. (XLS)

S1 PRISMA Checklist PRISMA checklist. (DOC)

S1 Table The frequency distribution of *NQO1* Pro187Ser for bladder cancer by smoking status. (DOC)

## References

[1] Jemal A, Bray F, Center MM, Ferlay J, Ward E, et al (2011) Global cancer statistics. CA Cancer J Clin 61: 69–90. 10.3322/caac.20107 21296855

[2] Bredberg A (2011) Cancer: more of polygenic disease and less of multiple mutations? A quantitative viewpoint. Cancer 117: 440–445. 10.1002/cncr.25440 20862743

[3] Pharoah PD, Dunning AM, Ponder BA, Easton DF (2004) Association studies for finding cancer-susceptibility genetic variants. Nat Rev Cancer 4: 850–860. 15516958 10.1038/nrc1476

[4] Parkin DM (2006) The global health burden of infection-associated cancers in the year 2002. Int J Cancer 118: 3030–3044. 16404738 10.1002/ijc.21731

[5] Lin BK, Clyne M, Walsh M, Gomez O, Yu W, et al (2006) Tracking the epidemiology of human genes in the literature: the HuGE Published Literature database. Am J Epidemiol 164: 1–4. 16641305 10.1093/aje/kwj175

[6] Menashe I, Figueroa JD, Garcia-Closas M, Chatterjee N, Malats N, et al (2012) Large-scale pathway-based analysis of bladder cancer genome-wide association data from five studies of European background. PLoS One 7: e29396 10.1371/journal.pone.0029396 22238607 PMC3251580

[7] Nioi P, Hayes JD (2004) Contribution of NAD(P)H:quinone oxidoreductase 1 to protection against carcinogenesis, and regulation of its gene by the Nrf2 basic-region leucine zipper and the arylhydrocarbon receptor basic helix-loop-helix transcription factors. Mutat Res 555: 149–171. 15476858 10.1016/j.mrfmmm.2004.05.023

[8] Guha N, Chang JS, Chokkalingam AP, Wiemels JL, Smith MT, et al (2008) NQO1 polymorphisms and de novo childhood leukemia: a HuGE review and meta-analysis. Am J Epidemiol 168: 1221–1232. 10.1093/aje/kwn246 18945694 PMC2727266

[9] Misra V, Grondin A, Klamut HJ, Rauth AM (2000) Assessment of the relationship between genotypic status of a DT-diaphorase point mutation and enzymatic activity. Br J Cancer 83: 998–1002. 10993645 10.1054/bjoc.2000.1359 PMC2363567

[10] Siegel D, McGuinness SM, Winski SL, Ross D (1999) Genotype-phenotype relationships in studies of a polymorphism in NAD(P)H:quinone oxidoreductase 1. Pharmacogenetics 9: 113–121. 10208650 10.1097/00008571-199902000-00015

[11] Golka K, Selinski S, Lehmann ML, Blaszkewicz M, Marchan R, et al (2011) Genetic variants in urinary bladder cancer: collective power of the "wimp SNPs". Arch Toxicol 85: 539–554. 10.1007/s00204-011-0676-3 21380501

[12] Schulz WA, Krummeck A, Rosinger I, Eickelmann P, Neuhaus C, et al (1997) Increased frequency of a null-allele for NAD(P)H: quinone oxidoreductase in patients with urological malignancies. Pharmacogenetics 7: 235–239. 9241663 10.1097/00008571-199706000-00008

[13] Choi JY, Lee KM, Cho SH, Kim SW, Choi HY, et al (2003) CYP2E1 and NQO1 genotypes, smoking and bladder cancer. Pharmacogenetics 13: 349–355. 12777965 10.1097/00008571-200306000-00006

[14] Park SJ, Zhao H, Spitz MR, Grossman HB, Wu X (2003) An association between NQO1 genetic polymorphism and risk of bladder cancer. Mutat Res 536: 131–137. 12694753 10.1016/s1383-5718(03)00041-x

[15] Hung RJ, Boffetta P, Brennan P, Malaveille C, Gelatti U, et al (2004) Genetic polymorphisms of MPO, COMT, MnSOD, NQO1, interactions with environmental exposures and bladder cancer risk. Carcinogenesis 25: 973–978. 14729580 10.1093/carcin/bgh080

[16] Moore LE, Wiencke JK, Bates MN, Zheng S, Rey OA, et al (2004) Investigation of genetic polymorphisms and smoking in a bladder cancer case-control study in Argentina. Cancer Lett 211: 199–207. 15219943 10.1016/j.canlet.2004.04.011

[17] Sanyal S, Festa F, Sakano S, Zhang Z, Steineck G, et al (2004) Polymorphisms in DNA repair and metabolic genes in bladder cancer. Carcinogenesis 25: 729–734. 14688016 10.1093/carcin/bgh058

[18] Broberg K, Bjork J, Paulsson K, Hoglund M, Albin M (2005) Constitutional short telomeres are strong genetic susceptibility markers for bladder cancer. Carcinogenesis 26: 1263–1271. 15746160 10.1093/carcin/bgi063

[19] Terry PD, Umbach DM, Taylor JA (2005) No association between SOD2 or NQO1 genotypes and risk of bladder cancer. Cancer Epidemiol Biomarkers Prev 14: 753–754. 15767364 10.1158/1055-9965.EPI-04-0574

[20] Figueroa JD, Malats N, Garcia-Closas M, Real FX, Silverman D, et al (2008) Bladder cancer risk and genetic variation in AKR1C3 and other metabolizing genes. Carcinogenesis 29: 1955–1962. 10.1093/carcin/bgn163 18632753 PMC2556968

[21] Wang YH, Lee YH, Tseng PT, Shen CH, Chiou HY (2008) Human NAD(P)H:quinone oxidoreductase 1 (NQO1) and sulfotransferase 1A1 (SULT1A1) polymorphisms and urothelial cancer risk in Taiwan. J Cancer Res Clin Oncol 134: 203–209. 17619904 10.1007/s00432-007-0271-4 PMC12161676

[22] Pandith AA, Khan NP, Shah ZA, Shah AM, Wani SM, et al (2011) Association of bladder cancer risk with an NAD(P)H:quinone oxidoreductase polymorphism in an ethnic Kashmiri population. Biochem Genet 49: 417–426. 10.1007/s10528-011-9418-8 21253823

[23] Basma HA, Kobeissi LH, Jabbour ME, Moussa MA, Dhaini HR (2013) CYP2E1 and NQO1 genotypes and bladder cancer risk in a Lebanese population. Int J Mol Epidemiol Genet 4: 207–217. 24319536 PMC3852640

[24] Fu J, Chen BC (2013) Relationship between genetic polymorphisms of CYP1A1, NQO1 and EPHX1 and susceptibility to bladder cancer. Chin J Cancer Prev Treat 20: 10–14.

[25] Goerlitz D, Amr S, Dash C, Saleh DA, El Daly M, et al (2014) Genetic polymorphisms in NQO1 and SOD2: interactions with smoking, schistosoma infection, and bladder cancer risk in Egypt. Urol Oncol 32: 47 e15–20. 10.1016/j.urolonc.2013.06.016 24035474 PMC3885358

[26] Huang ZM, Chen HA, Chiang YT, Shen CH, Tung MC, et al (2014) Association of polymorphisms in iNOS and NQO1 with bladder cancer risk in cigarette smokers. J Chin Med Assoc 77: 83–88. 10.1016/j.jcma.2013.10.005 24315573

[27] Mandal RK, Dubey S, Panda AK, Mittal RD (2014) Genetic variants of NQO1 gene increase bladder cancer risk in Indian population and meta-analysis. Tumour Biol. 24676794 10.1007/s13277-014-1869-1

[28] Fu J, Chen BC (2012) Relationship between genetic polymorphism of NQO1 and susceptibility to bladder cancer. Journal of Chinese Oncology 18: 561–564.

[29] Amr S, Dawson R, Saleh DA, Magder LS, George DM, et al (2013) Pesticide, Gene Polymorphisms and Bladder Cancer among Egyptian Agricultural Workers. Arch Environ Occup Health. 10.1080/19338244.2013.853646 24219772 PMC4018465

[30] Basu S, Brown JE, Flannigan GM, Gill JH, Loadman PM, et al (2004) NAD(P)H:Quinone oxidoreductase-1 C609T polymorphism analysis in human superficial bladder cancers: relationship of genotype status to NQO1 phenotype and clinical response to Mitomycin C. Int J Oncol 25: 921–927. 15375541

[31] Ryk C, Kumar R, Sanyal S, de Verdier PJ, Hemminki K, et al (2006) Influence of polymorphism in DNA repair and defence genes on p53 mutations in bladder tumours. Cancer Lett 241: 142–149. 16343742 10.1016/j.canlet.2005.10.025

[32] Sanyal S, Ryk C, De Verdier PJ, Steineck G, Larsson P, et al (2007) Polymorphisms in NQO1 and the clinical course of urinary bladder neoplasms. Scand J Urol Nephrol 41: 182–190. 17469025 10.1080/00365590600991946

[33] Higgins JP, Thompson SG, Deeks JJ, Altman DG (2003) Measuring inconsistency in meta-analyses. BMJ 327: 557–560. 12958120 10.1136/bmj.327.7414.557 PMC192859

[34] Mantel N, Haenszel W (1959) Statistical aspects of the analysis of data from retrospective studies of disease. J Natl Cancer Inst 22: 719–748. 13655060

[35] DerSimonian R, Laird N (1986) Meta-analysis in clinical trials. Control Clin Trials 7: 177–188. 3802833 10.1016/0197-2456(86)90046-2

[36] Egger M, Davey Smith G, Schneider M, Minder C (1997) Bias in meta-analysis detected by a simple, graphical test. BMJ 315: 629–634. 9310563 10.1136/bmj.315.7109.629 PMC2127453

[37] He J, Shi TY, Zhu ML, Wang MY, Li QX, et al (2013) Associations of Lys939Gln and Ala499Val polymorphisms of the XPC gene with cancer susceptibility: a meta-analysis. Int J Cancer 133: 1765–1775. 10.1002/ijc.28089 23400628

[38] Wacholder S, Chanock S, Garcia-Closas M, El Ghormli L, Rothman N (2004) Assessing the probability that a positive report is false: an approach for molecular epidemiology studies. J Natl Cancer Inst 96: 434–442. 15026468 10.1093/jnci/djh075 PMC7713993

[39] Yin ZB, Hua RX, Li JH, Sun C, Zhu JH, et al (2014) Smoking and hOGG1 Ser326Cys polymorphism contribute to lung cancer risk: evidence from a meta-analysis. Tumour Biol 35: 1609–1618. 10.1007/s13277-013-1222-0 24085357

[40] He J, Wang MY, Qiu LX, Zhu ML, Shi TY, et al (2013) Genetic variations of mTORC1 genes and risk of gastric cancer in an Eastern Chinese population. Mol Carcinog 52 Suppl 1: E70–79. 10.1002/mc.22013 23423739

[41] Chao C, Zhang ZF, Berthiller J, Boffetta P, Hashibe M (2006) NAD(P)H:quinone oxidoreductase 1 (NQO1) Pro187Ser polymorphism and the risk of lung, bladder, and colorectal cancers: a meta-analysis. Cancer Epidemiol Biomarkers Prev 15: 979–987. 16702380 10.1158/1055-9965.EPI-05-0899

[42] Guo ZJ, Feng CL (2012) The NQO1 rs1800566 polymorphism and risk of bladder cancer: evidence from 6,169 subjects. Asian Pac J Cancer Prev 13: 6343–6348. 23464456 10.7314/apjcp.2012.13.12.6343

[43] Zhang H, Wen X, Lu X (2013) Association between NAD(P)H:quinone oxidoreductase 1 rs1800566 polymorphism and risk of bladder cancer. Tumour Biol 34: 3377–3381. 10.1007/s13277-013-0909-6 23873104

[44] Gong M, Yi Q, Wang W (2013) Association between NQO1 C609T polymorphism and bladder cancer susceptibility: a systemic review and meta-analysis. Tumour Biol 34: 2551–2556. 10.1007/s13277-013-0799-7 23749485

[45] Lajin B, Alachkar A (2013) The NQO1 polymorphism C609T (Pro187Ser) and cancer susceptibility: a comprehensive meta-analysis. Br J Cancer 109: 1325–1337. 10.1038/bjc.2013.357 23860519 PMC3778271

[46] Malik MA (2014) Comment on ‘The NQO1 polymorphism C609T (Pro187Ser) and cancer susceptibility: a comprehensive meta-analysis’. Br J Cancer. 10.1038/bjc.2014.626 24736580 PMC4260013

[47] Dinkova-Kostova AT, Talalay P (2010) NAD(P)H:quinone acceptor oxidoreductase 1 (NQO1), a multifunctional antioxidant enzyme and exceptionally versatile cytoprotector. Arch Biochem Biophys 501: 116–123. 10.1016/j.abb.2010.03.019 20361926 PMC2930038

[48] Siegel D, Yan C, Ross D (2012) NAD(P)H:quinone oxidoreductase 1 (NQO1) in the sensitivity and resistance to antitumor quinones. Biochem Pharmacol 83: 1033–1040. 10.1016/j.bcp.2011.12.017 22209713 PMC3482497

[49] Benson AM (1993) Conversion of 4-nitroquinoline 1-oxide (4NQO) to 4-hydroxyaminoquinoline 1-oxide by a dicumarol-resistant hepatic 4NQO nitroreductase in rats and mice. Biochem Pharmacol 46: 1217–1221. 8216372 10.1016/0006-2952(93)90470-h

[50] Chen S, Knox R, Lewis AD, Friedlos F, Workman P, et al (1995) Catalytic properties of NAD(P)H:quinone acceptor oxidoreductase: study involving mouse, rat, human, and mouse-rat chimeric enzymes. Mol Pharmacol 47: 934–939. 7746280

[51] Boccia S, De Feo E, Galli P, Gianfagna F, Amore R, et al (2010) A systematic review evaluating the methodological aspects of meta-analyses of genetic association studies in cancer research. Eur J Epidemiol 25: 765–775. 10.1007/s10654-010-9503-z 20830507

