# Supplementary material for: The Association between NQO1 Pro187Ser Polymorphism and Bladder Cancer Susceptibility: A Meta-Analysis of 15 Studies
Source: PLoS One. 2015 Jan 20;10(1):e0116500. doi: 10.1371/journal.pone.0116500 (PMC4300190; doi:10.1371/journal.pone.0116500)
Supplement: S1 Table — (DOC) [file pone.0116500.s003.doc]

| **Supplemental Table 1.** The frequency distribution of *NQO1* Pro187Ser for bladder cancer by smoking status. | | | | | | | | |
| --- | --- | --- | --- | --- | --- | --- | --- | --- |
| Surname | Year | Smoking status | Case | | | Control | | |
|  |  |  | Pro/Pro | Pro/Ser | Ser/Ser | Pro/Pro | Pro/Ser | Ser/Ser |
| Park | 2003 | Ever | 104 |  | 62^a^ | 89 |  | 30^a^ |
| Park | 2003 | Never | 38 |  | 28^a^ | 74 |  | 46^a^ |
| Choi | 2003 | Ever | 40 |  | 46^a^ | 13 |  | 32^a^ |
| Choi | 2003 | Never | 35 |  | 37^a^ | 41 |  | 59^a^ |
| Moore | 2004 | Ever | 52 | 20 | 7 | 31 | 25 | 4 |
| Moore | 2004 | Never | 10 | 15 | 2 | 30 | 15 | 3 |
| Hung | 2004 | Ever | 107 | 66 | 11 | 99 | 53 | 9 |
| Hung | 2004 | Never | 6 | 9 | 2 | 36 | 13 | 4 |
| Terry | 2005 | Ever | 133 | 57 | 6 | 91 | 39 | 5 |
| Terry | 2005 | Never | 23 | 13 | 3 | 59 | 19 | 1 |
| Wang | 2008 | Ever | 33 |  | 114^a^ | 30 |  | 81^a^ |
| Wang | 2008 | Never | 37 |  | 116^a^ | 64 |  | 125^a^ |
| Pandith | 2011 | Ever | 34 |  | 46^a^ | 54 |  | 21^a^ |
| Pandith | 2011 | Never | 10 |  | 14^a^ | 25 |  | 20^a^ |
| Huang | 2014 | Ever | 21 | 44 | 22 | 18 | 21 | 11 |
| Huang | 2014 | Never | 15 | 39 | 18 | 33 | 46 | 21 |
| ^a^ The numbers were just given for *NQO1* Pro/Ser+Ser/Ser genotypes, only calculated for dominant model. | | | | | | | | |
